# The *CYP3A5* genotypes of both liver transplant recipients and donors influence the time‐dependent recovery of tacrolimus clearance during the early stage following transplantation

**DOI:** 10.1002/ctm2.542

**Published:** 2021-10-14

**Authors:** Li Huang, Abdullah A. Assiri, Peihao Wen, Kun Zhang, Junwei Fan, Tonghai Xing, Yuan Liu, Jinyan Zhang, Zhaowen Wang, Zhaojie Su, Jiajia Chen, Yi Xiao, Rui Wang, Risi Na, Liyun Yuan, Dehua Liu, Junjie Xia, Lin Zhong, Wanqing Liu, Wenzhi Guo, Brian R. Overholser, Zhihai Peng

**Affiliations:** ^1^ Department of General Surgery Shanghai General Hospital Shanghai Jiao Tong University Shanghai P. R. China; ^2^ Department of Clinical Pharmacy King Khalid University Abha Saudi Arabia; ^3^ Department of Pharmacy Practice College of Pharmacy Purdue University West Lafayette Indiana USA; ^4^ Department of Hepatobiliary and Pancreatic Surgery The First Affiliated Hospital Zhengzhou P. R. China; ^5^ Department of General Surgery School of Medicine Xiang'an Hospital of Xiamen University Xiamen University Xiamen Fujian P. R. China; ^6^ Organ Transplantation Institute School of Medicine Xiamen University Xiamen Fujian P. R. China; ^7^ Bio‐Med Big Data Center CAS‐MPG Partner Institute for Computational Biology Shanghai Institute of Nutrition and Health Chinese Academy of Sciences Shanghai Institutes for Biological Sciences Shanghai P. R. China; ^8^ Department of Pharmaceutical Sciences Eugene Applebaum College of Pharmacy and Health Sciences Wayne State University Detroit Michigan USA; ^9^ Division of Clinical Pharmacology Indiana University School of Medicine Indianapolis Indiana USA

Dear Editor,

The majority of allograft rejection occurs within 1 month after liver transplantation; with the highest incidence around 7–10 days. In this study, we demonstrate the impact of donor and recipient genotypes on tacrolimus clearance and dosing requirements during the first 28 days following liver transplantation. Tacrolimus is primarily metabolized by cytochrome P450 (CYP) 3A isozymes, CYP3A4 and CYP3A5, which mediate hepatic and intestinal biotransformation.^1^ However, it is unknown how the influence of *CYP3A5* genotype of the donor and recipient contribute to tacrolimus variability as liver performance improves with time in the early post‐operative phase.^2^, ^3^, ^4^, ^5^, ^6^, ^7^ There remains an unmet medical need to find an optimal dose regimen for immunosuppressants within the first few weeks after transplantation to avoid potential toxicities due to overdose or acute rejection.^2^ Thus, the goal of our work is to establish personalized immunosuppressive regimens following liver transplantation. By using genetics and patient‐related factors, individualized dosing regimens can be initiated and used with current drug monitoring protocols to decrease toxicity and graft rejection during the early phases of post‐transplant.

We enrolled adult patients in two independent cohorts undergoing orthotopic liver transplantation. Tacrolimus and mycophenolate mofetil were administered following transplant without steroids. Patients were excluded from undergoing multi‐organ transplantation or had incomplete data. Cohort A (index set) comprised 115 from Shanghai General Hospital Affiliated to Shanghai Jiao Tong University. Cohort B (validation set) comprised 95 patients from First Affiliated Hospital of Zhengzhou University. The patient demographics are displayed in Table 1. The research was carried out in accordance with the Declaration of Helsinki and was approved by the Ethics Committee of Shanghai General Hospital Affiliated to Shanghai Jiao Tong University and the First Affiliated Hospital of Zhengzhou University.

**TABLE 1:** Patient demographics

Characteristics	Index (n = 115)	Validation (n = 95)
Age, Yrs (mean ± SD)	47.5 ± 9	49 ± 9.6
Sex (F/M)	19/96	43/52
Weight, Kg (mean ± SD)	67.7 ± 10.9	65.5 ± 10.8
AST, u/L (mean ± SD)	133.9 ± 300	136.8 ± 527
ALT, u/L (mean ± SD)	104.7 ± 175.3	154.8 ± 348.3
HCT, (mean ± SD)	0.9 ± 7.2	2.3 ± 7.8
DBIL, μM (mean ± SD)	29.9 ± 42.3	42.7 ± 108.7
TBIL, μM (mean ± SD)	59.5 ± 74.5	50.4 ± 34.7
Hb, g/L (mean ± SD)	100.1 ± 18.1	100.3 ± 17.8
BUN, mM (mean ± SD)	7.1 ± 5.6	7.3 ± 7.7
Alb, g/L (mean ± SD)	38.2 ± 4.6	36.7 ± 4.6

CYP3A5 Genotype*	Donor/Recipient		
Expressers	E/E	26/115 (22.6%)	25/95 (26.3%)
Non‐expressers	NE/NE	28/115 (24.3%)	21/95 (22.1%)
Recipient‐Expressers	NE/E	27/115 (23.4%)	22/95 (23.1%)
Donor‐Expressers	E/NE	32/115 (27.8%)	26/95 (27.3%)

* *CYP3A5* genotype was not available in three recipients or donors.

Alb, Albumin; ALT, alanine aminotransferase; AST, aspartate aminotransferase; BUN, blood urea nitrogen; DBIL, direct bilirubin; DE, donor‐expresser; E, expresser; Hb, hemoglobin; NE, non‐expresser; RE, recipient‐expresser; TBIL, total bilirubin.

Tacrolimus (0.06–0.08 mg/kg/day) was administered twice daily for 28 days. Blood samples were collected prior to the morning administration. Tacrolimus was measured in whole blood by the Pro‐TracTMII tacrolimus ELISA kit (Diasorin, Stillwater, MN, USA) with a microparticle enzyme immunoassay (ELx 800NB analyser, BioTek, Winooski, VT, USA). DNA was isolated from both recipients’ and donors’ liver tissue using an AllPrep DNA/RNA Mini Kit (Qiagen, Hilden, Germany). *CYP3A5* rs776746 were genotyped using real‐time PCR.

A population pharmacokinetic (PK) analysis was performed using the ADVAN4 TRANS4 subroutine of NONMEM version 7 (ICON Development Solutions, Ellicott City, MD, USA). The PK parameters and within‐ and between‐subject variability were estimated using first‐order conditional estimation with interaction. A two‐compartment model with first‐order absorption adequately described the data. Inter‐individual and residual variability were best described by the proportional error model. All population parameter estimates are summarized in Table 2.

**TABLE 2:** PK parameters estimates

Parameter	Base model estimate	Final model estimate	Bootstrap, Cl (2.5, 97.5%)
CL/F (L/h)	13.7	–	
Non‐expressers CL/F (L/h)	–	7.56	7.6 (6.5,8.5)
Expressers CL/F (L/h)	–	13.3	13.26 (11,16.5)
Recipient‐expressers CL/F (L/h)	–	9.59	9.56 (8.1,10.7)
Donor‐expressers CL/F(L/h)	–	10.1	10.1 (9,11.4)
Vc/F(L)	182	245	241.8 (139.6,365.3)
Q/F (L/h)	78*	78*	78*
Vp/F(L)	327*	327*	327*
K_a_ (h^−1^)	0.473*	0.473*	0.473*
POD on CL/F	–	0.57	0.56 (0.45,0.66)
Dose on CL/F	–	2.26	2.29 (1.85,2.89)
*ω* CL/F (%)	57	35	34.6 (28.3,41.2)
*ω* Vc/F(%)	138	147	147 (115.3,180)
*ω* Vp/F (%)	30*	30*	30*
*σ*	0.319	0.164	0.163 (0.15,0.18)

* The Q/F–Inter‐compartmental clearance following oral dose, absorption rate constant (*Ka*), and peripheral volume (Vp/F) were fixed to 78 L/h, 0.473/h, and 327 L, respectively.^10^

CL/F, Clearance following oral dose; POD, post‐operative days; *σ*, residual error; Vc/F, central volume of distribution following oral dose; ω, between subject variability.

Potential covariates were identified by generalized additive model using Xpose package in R. The covariates were included if the difference of objection function value (ΔOFV; model evaluation measure) was more than 3.84 by forward inclusion. Covariates were removed from final model if ΔOFV is more than 7.88, α = 0.05 by stepwise backward elimination. Additive, proportional and exponential inter‐individual variability structure models for continuous covariates, and binary string structure model for categorical covariates were tested. Each covariate was tested independently; Supporting information Table S1. The final model included post‐operative days (POD), dose, and combined genotype as significant predictors of tacrolimus clearance; Supporting information Table S2.

The individual and population predictions of the measured concentrations and visual predictive checks (VPC) are displayed in Figure 1. The parameter estimates in the final model were comparable to the median parameter estimates obtained from bootstrapping and fell within the 95% CL (Table 2) indicating acceptable precision and stability of the parameter estimations from the final model. The adequacy of model prediction was assessed by mean precision error (MPE) and mean absolute precision error (MAPE) for tacrolimus concentrations in the external validation cohort; Supporting information Table S3. The overall bias (MPE) was 0.19 ng/mL (95% CI −0.16, 0.35) and MPE% was 18.8%; comparable to the proportional residual error in the final model of 16.4%. The precision (MAPE) was 2.13 ng/mL (MAPE = 39%). Both MPE and MAPE were not significantly different from zero.

**FIGURE 1:**
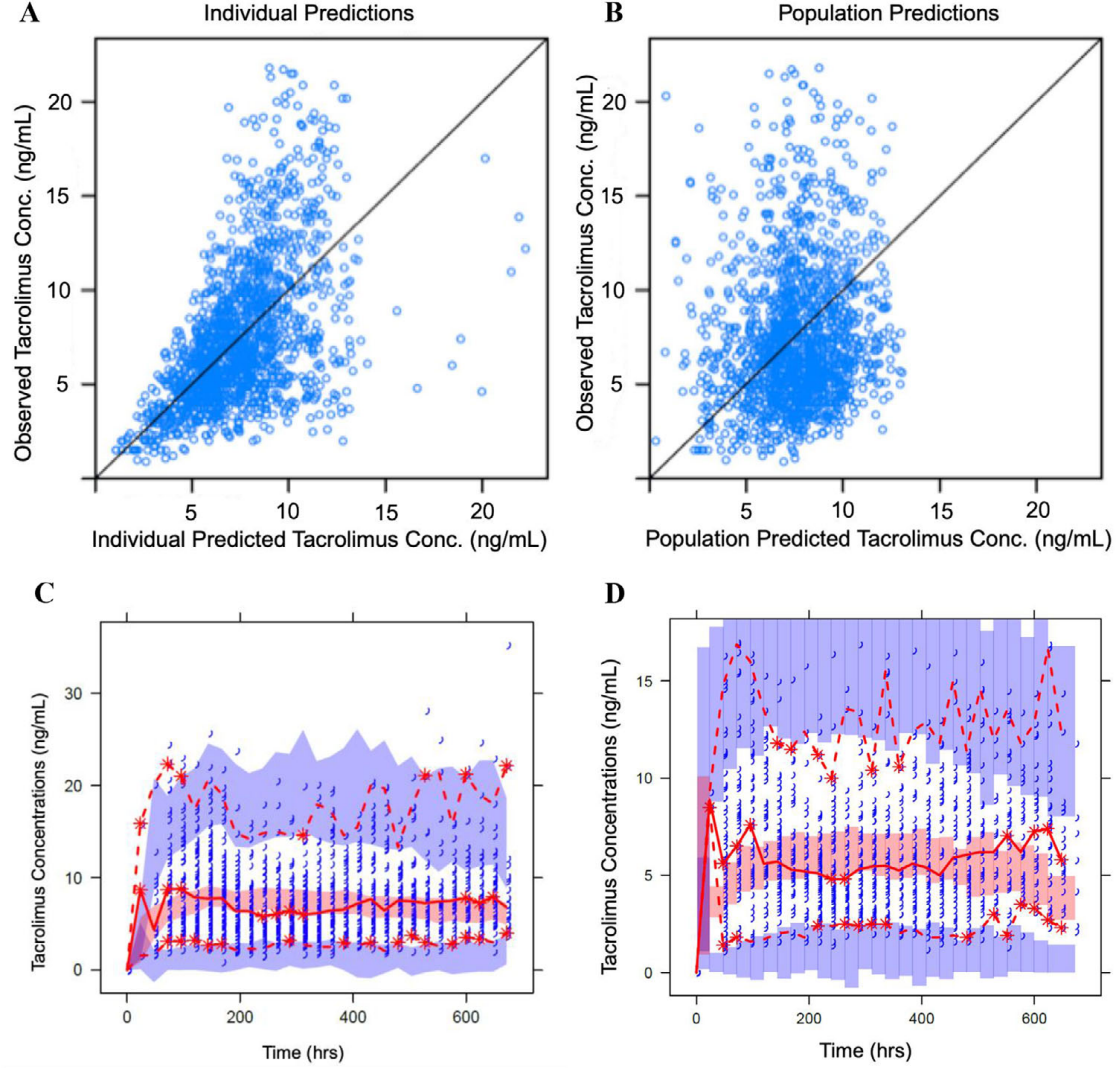
Goodness‐of‐Fit plots for the final model: (A) Observed tacrolimus blood concentrations versus individual model predictions. (B) Observed tacrolimus blood concentrations versus population model predictions. The solid line represents the line of identity. (C) VPCs of the final model (index cohort). The middle solid line (red) represents the median of the observed data where the red shadow area represents the corresponding model‐based confidence intervals. The dashed red lines are 2.5 and 97.5% while the blue fields are the corresponding model‐based confidence intervals. The observed data were in accordance with the model‐based confidence intervals and most of the observed data were inside the 95% confidence interval indicating the model adequately predicted the trough concentrations. (D) VPCs of the final model in the external dataset (validation cohort). The middle solid line (red) represents the median of the observed data where the red shadow area represents the corresponding model‐based confidence intervals. The dashed red lines are 2.5 and 97.5% while the blue fields are the corresponding model‐based confidence intervals. The observed data were in accordance with the model‐based confidence intervals and most of the observed data were inside the 95% confidence interval indicating the model adequately predicted the trough concentrations

Tacrolimus population clearance increased over the course of the first 28 days following transplantation; Figure 2A and B. The gradual increase in clearance is likely due to the stabilization of liver function with POD following transplantation. The level of increase in CL/F in tacrolimus was dependent on *CYP3A5* genotypes of donors and recipients with a greater than threefold increase in combined donor and recipient CYP3A5 expressers. CL/F was significantly higher in CYP3A5 expressers when compared to CYP3A5 non‐expressers, 13.3 ± 1.0 versus 7.7 ± 0.6 L/h (*p* < 0.0001). The CYP3A5 expressers group CL/F was also higher than both mixed donor or recipient expressers, 13.3 ± 1.0 versus 9.3 ± 0.7 or 9.8 ± 0.7, respectively (*p* < 0.05).

**FIGURE 2:**
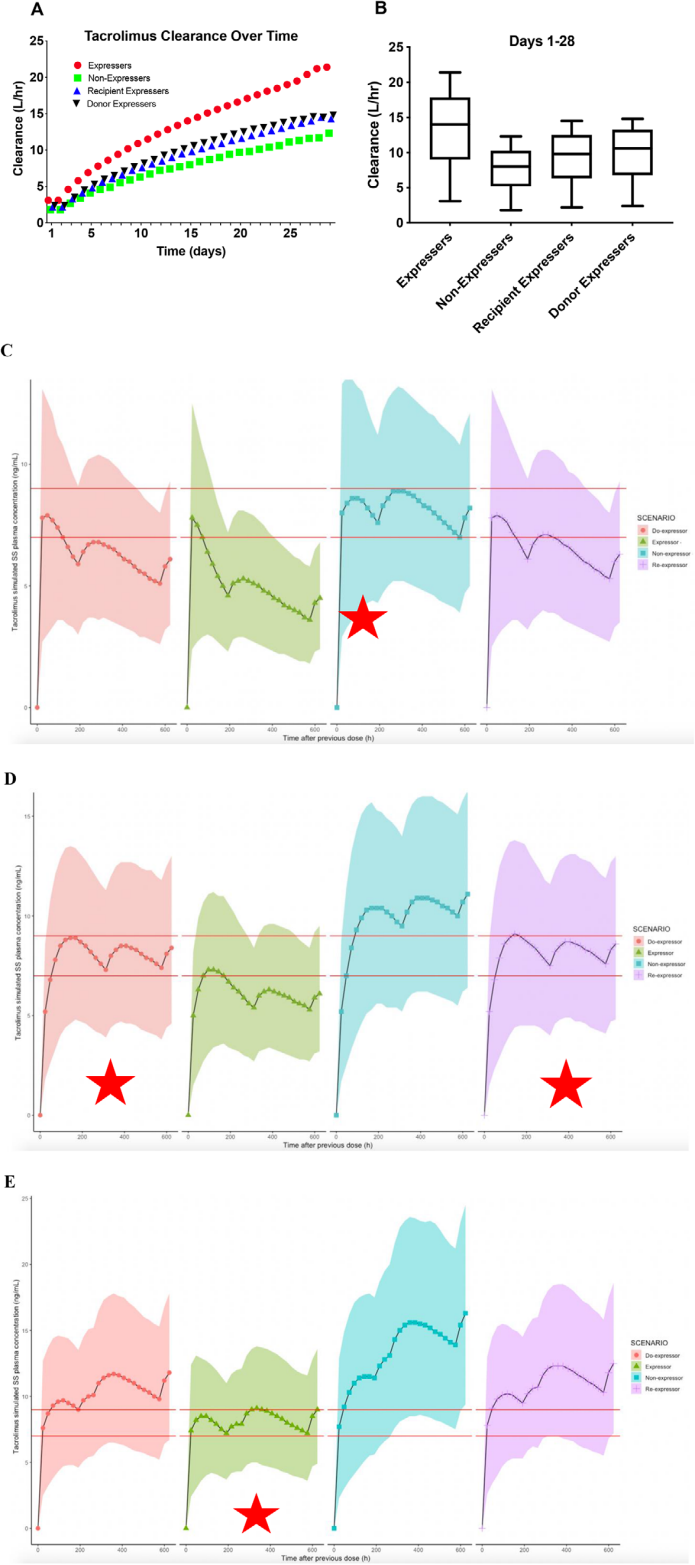
The clearance of tacrolimus increases with post‐operative days for the first 28 days following liver transplantation. (A) Population tacrolimus CL/F at each day for 28 days following liver transplant in each genotype group. (B) The overall mean tacrolimus CL/F for 28 days combined for each genotype group. (C) Simulation of tacrolimus oral dosing of 0.075/mg/kg/day (day 1); 0.0125/mg/kg/day (day 2–7); 0.025/mg/kg/day (day 8–23); 0.0375/mg/kg/day (day 24–28). This adaptive dosing regimen was most appropriate for the combined recipient and donor CYP3A5 non‐expressers (blue) for 28 days following liver transplantation. (D) Simulation of tacrolimus oral dosing of 0.05/mg/kg/day (day 1); 0.025/mg/kg/day (day 2–12); 0.375/mg/kg/day (day 13–23);0.05/mg/kg/day (days 24–28). This adaptive dosing regimen was most appropriate for both Donor (red) and Recipient (purple) expressers combined with non‐expressers for 28 days following liver transplantation. (E) Simulation of tacrolimus oral dosing of 0.075/mg/kg/day (day 1); 0.025/mg/kg/day (day 2–7); 0.375/mg/kg/day (day 8–10); 0.05/mg/kg/day (day 11–23); 0.075/mg/kg/day (day 24–28). This adaptive dosing regimen was most appropriate for the combined recipient and donor CYP3A5 expressers (green) for 28 days following liver transplantation

The developed model was used to simulate dosing regimens to achieve tacrolimus blood concentrations of 7–9 ng/mL for each *CYP3A5* genotype combination. The covariate model‐based simulations (n = 1000 per *CYP3A5* genotype group per dosing regimen) was performed and different dosing regimens were calculated based on patients’ average body weight (67. 7 kg). Given the improvement of liver function dependence on *CYP3A5* genotype in the first 28 days post‐transplant, standardized tacrolimus dosing regimens did not maintain the desired target concentrations. Therefore, adaptive dosing regimens were optimized with the goals to (1) maintain trough tacrolimus concentrations between 7 and 9 ng/mL; (2) maintain 95% of the population tacrolimus concentrations above 5 ng/mL and below 20 ng/mL and (3) minimize the number of dosing changes. The tacrolimus trough blood concentrations are displayed for the optimal dosing regimen in CYP3A5 non‐expressers (Figure 2C); donor‐expressers and recipient‐expressers (Figure 2D); and combined donor and recipient expressers (Figure 2E) following tacrolimus oral dosing.

The optimal administration of tacrolimus in the early stage of recovery following liver transplantation is critical for controlling toxicity and patients’ long‐term prognosis.^1^ However, the optimal dosing strategy at this stage is unclear.^2^, ^8^, ^9^ In this study, we developed a population model and simulated adaptive dosing regimens for tacrolimus in these critical first 28 days after liver transplantation. The final model demonstrates that tacrolimus CL/F is on a significantly different trajectory in the first 28 days post‐transplantation depending on both the recipient and donor genotype. Given the *CYP3A5* genotype frequency among Chinese and other East and South Asian populations, this study highlights the importance of pharmacogenomics, drug monitoring, and adaptive dosing regimens for tacrolimus.

## CONFLICTS OF INTEREST

The authors declare no conflict of interest.

## FUNDING

This work was supported by the National Natural Science Foundation of China (Grant Number 81530044).

## Supporting information

Supporting information
