# Supplementary material for: The CYP3A5 genotypes of both liver transplant recipients and donors influence the time‐dependent recovery of tacrolimus clearance during the early stage following transplantation
Source: Clin Transl Med. 2021 Oct 14;11(10):e542. doi: 10.1002/ctm2.542 (PMC8516335; doi:10.1002/ctm2.542)
Supplement: Supplementary file 1 — Supporting information [file CTM2-11-e542-s001.docx]

**Supplemental Table 1: Covariate analysis**

| **Model** | **OFV** | **Δ OFV** | ***P* value** |
| --- | --- | --- | --- |
| **Base model** | 5247 |  |  |
| **POD on CL/F** | 4272 | -975 | <0.05 |
| **Dose on CL/F** | 4503 | -744 | <0.05 |
| **TBIL on CL/F** | 4899 | 348 | <0.05 |
| **DBIL on CL/F** | 5006 | -241 | <0.05 |
| **BUN on CL/F** | 5019 | -228 | <0.05 |
| **ALT on CL/F** | 5165 | -82 | <0.05 |
| **Combined Genotyping on CL/F** | 5223 | -24 | <0.05 |
| **AST on CL/F** | 5225 | -22 | <0.05 |
| **Hb on CL/F** | 5241 | -6 | <0.05 |
| **ALB on CL/F** | 5241 | -6 | <0.05 |
| **Age on CL/F** | 5245 | -2 | ns |
| **Sex on CL/F** | 5246 | -1 | ns |
| **HCT on CL/F** | 5246 | -1 | ns |
| **Weight on CL/F** | 5246 | -1 | ns |
| **Weight on V/F** | 5247 | 0 | ns |
| **POD + Dose on CL/F** | 3972 | -300 | <0.05 |
| **POD + Dose + TB on CL/F** | 4135 | 163 | ns |
| **POD + Dose + DB on CL/F** | 4103 | 131 | ns |
| **POD + Dose + BUN on CL/F** | 4104 | 132 | ns |
| **POD + Dose + ALT on CL/F** | 4081 | 109 | ns |
| **POD + Dose + Combined genotyping on CL/F** | 3957 | -15 | <0.05 |
| **POD + Dose + Combined genotyping + AST on CL/F** | 4066 | 109 | ns |
| **POD + Dose + Combined genotyping + Hg on CL/F** | 4142 | 185 | ns |
| **POD + DOSE + Combined genotyping + ALB on CL/F** | 4154 | 197 | ns |
| **Dose + Combined genotyping on CL/F** | 4454 | 497 | ns |
| **POD + Combined genotyping on CL/F** | 4243 | 286 | ns |

OFV – Objective Function Value; CL/F – Clearance following oral dose; POD – Post Operative Days; TBIL – Total Bilirubin; DBIL – Direct Bilirubin; BUN – Blood Urea Nitrogen; ALT - Alanine Aminotransferase; AST - Aspartate Aminotransferase; Hb – Hemoglobin; Alb – Albumin: HCT – Hematocrit

**Supplemental Table 2: Change of objective function value of covariate analysis**

| **Change of objective function value of covariate analysis** | | | |
| --- | --- | --- | --- |
|  | **OFV** | **ΔOFV** | **𝑃 value** |
| **Inclusion** |  |  |  |
| **Base model** | 5247 | - | - |
| **Influence of POD on CL/F** | 4272 | -975 | <0.05 |
| **Influence of dose on CL/F** | 4503 | -744 | <0.05 |
| **Influence of genotyping on CL/F** | 5223 | -24 | <0.05 |
|  |  |  |  |
| **Elimination** |  |  |  |
| **Full model** | 3957 | - | - |
| **Elimination genotyping on CL/F** | 3972 | 24 | <0.01 |
| **Elimination dose on CL/F** | 4243 | 286 | <0.01 |
| **Elimination POD on CL/F** | 4454 | 497 | <0.01 |

OFV – Objective Function Value; CL/F – Clearance following oral dose; POD – Post Operative Days

**Supplemental Table 3: Prediction performance of final model in an independent validation dataset**

| **Tacrolimus (ng/mL)** | **Bias (MPE%)** | **95% CI** | **Imprecision (MAPE%)** | **95% CI** |
| --- | --- | --- | --- | --- |
| **All 28 Days** | 18.8 | (-3.0, 22) | 39.7 | (-2.4, 42) |
| **Day 1** | 4.2 | (-12, 7.7) | 39.2 | (-8.8, 48) |
| **Day 4** | 1.1 | (-12, 13) | 29.1 | (-8.7, 38) |
| **Day 15** | 25.8 | (-15, 41) | 36.3 | (-13, 49) |
| **Day 20** | 9.4 | (-13, 22) | 32.8 | (-9, 42) |
| **Day 26** | 7.4 | (-12, 4.4) | 29.0 | (-6.3, 35) |

MPE% - Mean Percentage Error; MAPE% - Mean Absolute Percentage Error; CI – Confidence Interval
